# Osteopenia is associated with inferior survival in patients undergoing partial hepatectomy for hepatocellular carcinoma

**DOI:** 10.1038/s41598-022-21652-z

**Published:** 2022-10-31

**Authors:** Franziska Alexandra Meister, Suekran Verhoeven, Anna Mantas, Wen-Jia Liu, Decan Jiang, Lara Heij, Daniel Heise, Philipp Bruners, Sven Arke Lang, Tom Florian Ulmer, Ulf Peter Neumann, Jan Bednarsch, Zoltan Czigany

**Affiliations:** 1 Department of Surgery and Transplantation, Faculty of Medicine, University Hospital RWTH Aachen, Aachen, Germany; 2 Present Address: Department of Surgery, Charité-Universitätsmedizin Berlin, Campus Charité Mitte | Campus Virchow-Klinikum; Augustenburger Pl. 1, 13353 Berlin, Germany; 3 Institute of Radiology, Faculty of Medicine, University Hospital RWTH Aachen, Aachen, Germany; 4 Department of Surgery, Maastricht University Medical Centre (MUMC), Maastricht, The Netherlands

**Keywords:** Liver cancer, Hepatocellular carcinoma

## Abstract

Osteopenia is known to be associated with clinical frailty which is linked to inferior outcomes in various clinical scenarios. However, the exact prognostic value of osteopenia in patients undergoing curative intent-surgery for hepatocellular carcinoma (HCC) is not completely understood. This retrospective study was conducted in a cohort of 151 patients who underwent partial hepatectomy for HCC in curative intent at a German university medical center (05/2008–12/2019). Preoperative computed tomography-based segmentation was used to assess osteopenia, and the prognostic impact of pathological changes in bone mineral density (BMD) on perioperative morbidity, mortality, and long-term oncological outcome was analyzed. Five-year overall survival of osteopenic patients was significantly worse compared to those with normal BMD (29% vs. 65%, *p* = 0.014). In line with this, the probability of disease-free survival at 5 years was significantly worse for patients with osteopenia (21% vs. 64%, *p* = 0.005). In our multivariable model, osteopenia was confirmed as an independent risk-factor for inferior overall survival (Hazard-ratio 7.743, *p* = 0.002). Concerning perioperative complications, osteopenic patients performed slightly worse, even though no statistical difference was detected (Clavien-Dindo ≥ 3b; 21% vs. 9%, *p* = 0.139). The present study confirms osteopenia as an independent risk-factor for inferior survival in patients undergoing partial hepatectomy for HCC in a European cohort. Further studies are warranted to validate these findings.

## Introduction

Hepatocellular carcinoma (HCC) has become one of the leading causes of cancer-related death around the globe^1^. With respect to the central role of the liver in metabolism, most HCC patients are at high-risk of developing pathological alterations of body composition (BC), due to the underlying chronic liver disease^2^.

Over the past decade, impairment of BC, including depletion of muscle mass (sarcopenia) as well as muscle quality (myosteatosis) have been found to affect perioperative outcomes in various clinical conditions^3–5^. Previous studies have shown a strong association of sarcopenia with poor overall survival (OS) in patients undergoing liver resection for HCC^6–8^ and recent studies conducted by our group detected not only a high prevalence of myosteatosis, but also an association between myosteatosis and poor perioperative outcomes in patients undergoing orthotopic liver transplantation (OLT)^9,10^. Reduced bone mineral density (BMD), defined as osteopenia, is the most important factor of bone fragility^11^. Although Dual-energy X-ray absorptiometry (DXA) is the gold standard in examining BMD, CT scan-based attenuation values are increasingly used to characterize BMD, due to its broad availability in oncological patients as part of the pre-operative oncological staging^12^. Osteopenia is also associated with frailty^13^, and according to the data of Pereira et al., bone loss may even begin and become clinically detectable before reduction of skeletal muscle mass in patients suffering from chronic diseases^14^. Recently, studies from Asian cohorts have demonstrated the prognostic value of BMD in the context of mortality in HCC patients undergoing partial hepatectomy or OLT^15,16^.

Based on the above-mentioned information, the aim of this study was to analyze the prognostic role of BMD in clinical outcomes in a Western-European single-center cohort of HCC patients undergoing partial hepatectomy in curative intent.

## Patients and methods

### Patients and eligibility

All consecutive patients who underwent partial hepatectomy for HCC at the University Hospital RWTH Aachen (UH-RWTH), Aachen, Germany, between May 2008 and December 2019 were considered for inclusion into this retrospective analysis. Clinical staging was performed prior to elective surgery and patients with systemic or irresectable disease were excluded. Patients where the abdominal staging was performed by MRI were not eligible for the analysis of BMD and therefore have been excluded. The present study was carried out in accordance with the principles of the current version of the Declaration of Helsinki and the good clinical practice (ICH-GCP). The protocol has been approved by the RWTH-Aachen University Institutional Review Board (EK 115/20 and EK 341/21). The IRB ("Ethik-Kommission der RWTH Aachen") waived informed consent due to the retrospective study design and collection of routine clinical data.

### Image analysis and segmentation

Bone mineral density (BMD) was determined using imaging data as described by Sharma et al. using a single cross-sectional image at the level of 11th thoracic vertebra^15^. Up to 12 weeks prior to partial hepatectomy, a computed tomography was performed at the UH-RWTH Aachen for oncological staging. Technical data for CT image acquisition were chosen as the following: 128-section CT scan (SOMATOM Definition Flash, Siemens Healthcare, Erlangen, Germany) with 128 × 0.6 mm section collimation, a gantry rotation time of 0.5 s, a tube potential of 120 kV or a 40-section CT scan (SOMATOM Definition AS, Siemens Healthcare, Erlangen, Germany).

**Figure 1:**
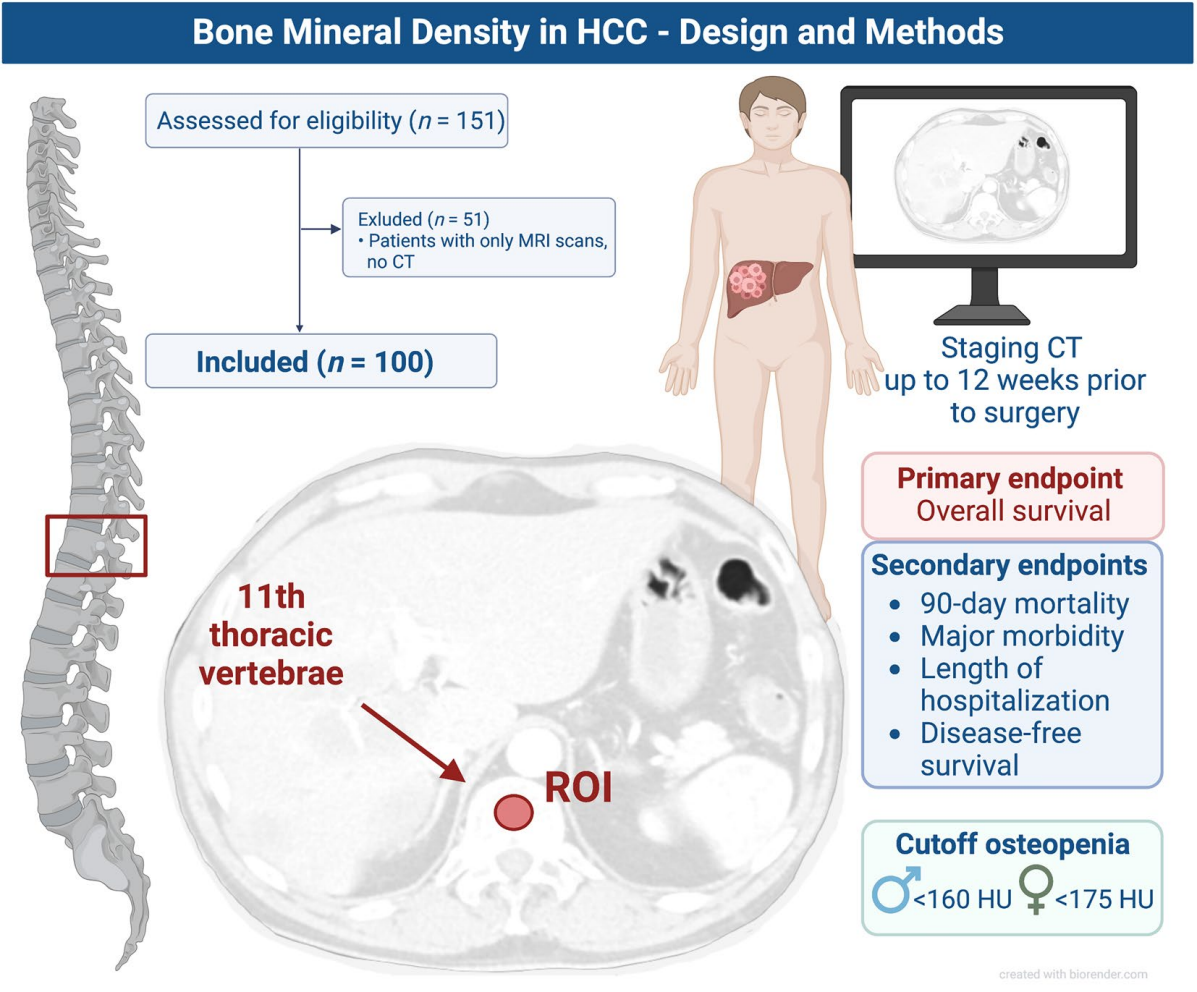
Abbreviations used: HCC: hepatocellular carcinoma; CT-computed tomography; ROI: region of interest; HU: Hounsfield units. This figure was created using BioRender.com.

An experienced investigator, who was blinded for the remaining clinical data of the patients, conducted the segmentation in a semi-automated fashion. Briefly, the average pixel density within a single standardized click-and-drag circular region of interest (ROI) defined as the mid-vertebral core sample on the trabecular bone of the 11th thoracic vertebra alone was calculated for all patients using the non-contrast plain phase of the CT scans (Fig. 1)^12^. To avoid incorrect measurements imaging-related artifacts or regions including the venous plexus have been avoided. Bone mineral density values are displayed in Hounsfield units (HU) where lower attenuation values are associated with poorer bone density.

In this particular study, due to the relatively small cohort and lower event numbers, we decided against the use of newly defined and not validated cutoffs based on the area under the curve analysis and the Youden-index, as it was described by our group in multiple previous reports^10,17–21^. Therefore, we utilized a previously described and established cut-off value of < 160 HU for male HCC patients based on Sharma et al.^15^ (Fig. 1). Further, as the distribution of BMD was statistically significant between females and males in our cohort, we defined a cut-off of < 175 HU for females based on their cohort-specific median value to adjust for the gender-specific differences in BMD described before^22^ (Fig. 1). Further body composition parameters related to the muscle and fat compartments including skeletal muscle index (SMI), visceral fat area (VFA), subcutaneous fat area (SFA) and visceral-to-subcutaneous fat ratio (VSR) were also assessed and reported as described previously^5,8,10,20,21^.

### Clinical data collection and patient follow-up

All clinical data were collected in a prospectively maintained institutional database and analyzed retrospectively. Indication for curative-intent partial hepatectomy was made by a staff hepatobiliary surgeon which was then confirmed by the institutional interdisciplinary tumor board. The partial hepatectomy was performed either laparoscopic or conventionally. Techniques of liver resection including the exact method of parenchymal transection were described by our group in previous studies ^23–25^. The outpatient clinic of the UH-RWTH Aachen as well as the local community based hepatologist network provided the follow-up data used in this study.

Classifications and scores reported in this analysis have been described by our group and by others in previous published studies (including ASA, labMELD, Clavien-Dindo classification-CD and the Comprehensive Complication Index-CCI^19,26–28^, procedural costs^29^, calculation of transfusion, of the length of hospital stay^9,19,30^ and long-term follow-up^21^).

### Statistical analysis

The primary endpoint of this study was defined as overall survival (OS) of patients undergoing liver resection for HCC. The incidence of perioperative in-hospital major morbidity (defined by CD ≥ 3b)^26^, overall perioperative outcome, length of hospital-stay, 90-day mortality, and disease-free survival (DFS) were analyzed and reported as secondary endpoints. Categorial data was reported as absolute and relative frequencies and continuous data were displayed as mean ± standard deviation. Where appropriate, the Chi-square test and Fisher's exact test were used to analyze categorical data. The Student t test, Mann–Whitney U test, and Kruskal–Wallis H test were used to analyze continuous data. Spearman correlation coefficient was used to further analyze the association of BMD and various BC parameter. The associations of survival with BC characteristics were assessed using uni- and multivariable Cox proportional hazards regression models. Survival curves were generated by the Kaplan–Meier method and compared with the log-rank test. Statistical analysis has been performed using SPSS Statistics 24 (IBM Corp., Armonk, NY, USA) and the level of statistical significance was set to *p* < 0.05.

## Results

### Study population characteristics

During the defined study period, 151 consecutive patients underwent curative-intend liver surgery for HCC at our institution. Some 51 patients were excluded due to insufficient preoperative imaging which yielded a final study cohort of 100 patients inculding 72 male (72%) and 28 female (28%) patients with a mean age of 67 ± 11 years. Histological cirrhosis has been confirmed in 42 patients and the mean preoperative labMELD was 8 ± 3. Prior to surgery, 22 patients were within the Milan criteria. Some 67 patients were categorized as performance status ASA III or higher and 71 patients suffered from HCC classified as UICC category I or II (n = 36, 35, respectively). A total of 38 patients suffered from more than one intrahepatic tumor. The mean largest tumor diameter was 72 ± 41 mm and the mean number of tumors was 1.9 ± 1.3, retrospectively. Hemihepatecomy (25%) and bisegmentectomy (25%) were the most frequently used operative procedures, followed by atypical resections (24%). In 21% of the cohort, laparoscopic procedure has been performed and R0 resection was achieved in 85% of patients (Table 1).

**Table 1:** Patient and procedural characteristics.

Characteristics	All patients	Osteopenia		*p* value
	n = 100	no n = 33	yes n = 67	
Patient age (years)	67 ± 11	61 ± 14	70 ± 9	**0.000**
Patient BMI	26 ± 4	25 ± 4	26 ± 5	0.359
Patient sex ratio (F:M)	28:72	9:24	19:48	0.909
**ASA**				
1	2	1 (3)	1 (2)	0.606
2	33	12 (36)	21 (31)	0.616
3	59	20 (61)	39 (58)	0.819
4	6	0 (0)	6 (9)	0.076
Preoperative labMELD	8 ± 3	8 ± 2	8 ± 3	0.180
Milan criteria	22	10 (30)	12 (18)	0.141
Cirrhosis	42	15 (46)	27 (40)	0.536
Preoperative AFP (µg/l)	2400 ± 9735	2787 ± 12,312	2141 ± 7787	0.263
Preoperative platelets (G/l)	247 ± 119	261 ± 144	238 ± 105	0.628
Preoperative AST (U/l)	54 ± 37	52 ± 40	55 ± 35	0.359
Preoperative ALT (U/l)	47 ± 46	56 ± 60	42 ± 33	0.601
Preoperative GGT (U/l)	170 ± 164	147 ± 145	180 ± 171	0.341
Preoperative albumin (g/l)	38 ± 10	37 ± 10	38 ± 9	0.680
SMI (cm^2^/m^2^)	45 ± 9	47 ± 9	45 ± 9	0.479
VFA (cm^2^)	174 ± 118	139 ± 90	191 ± 126	**0.070**
SFA (cm^2^)	188 ± 86	182 ± 88	191 ± 86	0.536
VSR	0.98 ± 0.67	0.85 ± 0.56	1.04 ± 0.73	0.273
BMD (HU)	153 ± 53	213 ± 40	123 ± 26	**0.000**
**Preoperative therapie**				
PVE	6	0 (0)	6 (9)	0.076
Sorafenib	1	0 (0)	1 (2)	0.481
TACE	7	3 (9)	4 (6)	0.565
SIRT	3	0 (0)	3 (5)	0.217
**Operative procedure**				
Atypical	24	11 (33)	13 (19)	0.125
Segmentectomy	20	5 (15)	15 (22)	0.395
Bisegementectomy	6	3 (9)	3 (5)	0.361
Hemihepatectomy	25	7 (21)	18 (27)	0.539
Extended resection	25	5 (15)	15 (22)	0.395
ALPPS resection	2	1 (3)	1 (2)	0.606
Other	3	1 (3)	2 (3)	0.990
Laparoscopic Procedure	21	8 (24)	13 (19)	0.524
**Tumor Stage UICC**				
I	36	15 (46)	20 (30)	0.124
II	35	11(33)	24 (36)	0.806
IIIa	18	3 (9)	15 (22)	0.104
IIIb	5	0 (0)	5 (8)	0.107
IIIc	2	1 (3)	1 (2)	0.606
IVa	3	1 (3)	2 (3)	0.990
IVb	1	1 (3)	0 (0)	0.152
Largest Tumor Diameter (mm)	72 ± 41	65 ± 34	76 ± 44	0.289
Number of Tumors	1.9 ± 1.3	1.6 ± 1.3	2 ± 1.4	**0.030**
R0 Resection	85	28 (85)	57 (85)	0.746

Values were given as mean ± standard deviation or absolute and relative frequencies (per cent). ^1^Refers to Clavien et al.^26^ ^3^Refers to Slankamenac et al.^27^ ^4^Refers to Staiger et al.^29^.

*BMI* Body mass index; *ASA* American Society of Anesthesiologists; *MELD* model for end-stage liver disease; *AFP* alphafetoprotein; *AST* Aspartate aminotransferase; *ALT* Alanine aminotransferase; *GGT* Gamma glutamyltransferase; *SMI* Sceletal muscle index; *VFA* Visceral fat area; *SFA* Subcutanous fat area, *VSR* Visceral-to-subcutaneous fat ratio; *BMD* (*HU*) Bone mineral density (Hounsfield units); *PVE* Portal venous embolization; *TACE* Transarterial chemoembolization; *SIRT* Selective internal radiotherapy; *UICC* Union for International Cancer Control.

Significant values are in bold.

### Body composition assessment

The median time between the CT imaging used for segmentation and surgery was 19 [6–47] days. In our cohort, the mean BMD was 153 ± 53 HU with a mean BMI of 26 ± 4. The mean SMI, a parameter to characterize muscle mass and sarcopenia, was 45 ± 9 cm^2^/m^2^ for all included patients.

**Figure 2:**
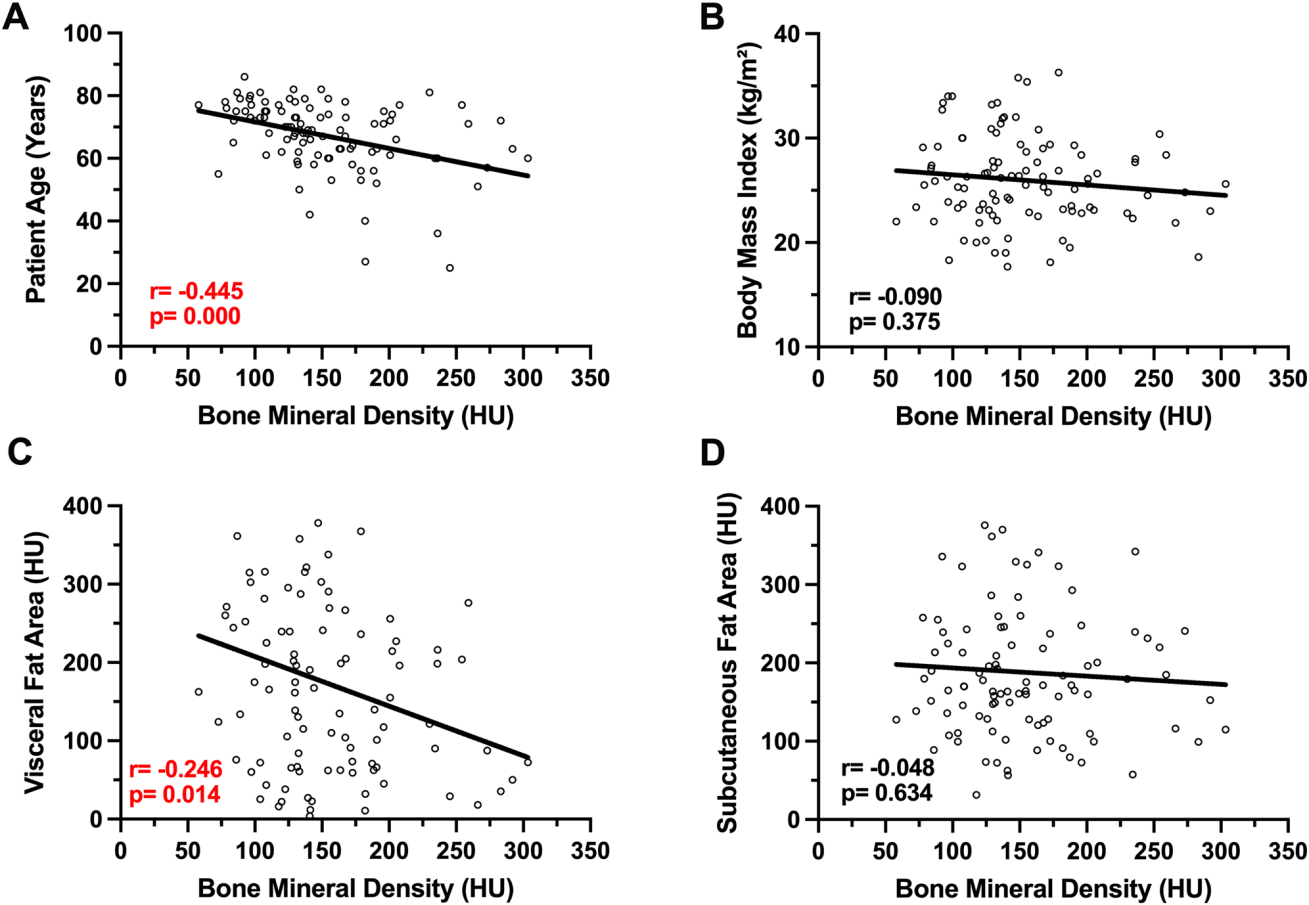
Correlation between bone mineral density and patient age (**A**), body mass index (**B**), visceral fat area (**C**) and subcutaneous fat area (**D**).

Concerning demographics and clinical characteristics, osteopenic patients were significantly older than non-osteopenic patients (70 ± 9 vs. 61 ± 14 years; *p* < 0.001, Table 1) and presented with a higher number of tumor nodules (2 ± 1.4 vs. 1.6 ± 1.3; *p* = 0.030, Table1). While BMI and SMI did not differ between groups (*p* = 0.359; *p* = 0.479, Table 1), muscle quality (L3Muscle-RA) was significantly inferior in osteopenic patients (31 ± 10 vs. 36 ± 9 HU; *p* = 0.049, Table 1) and the amount of visceral fat (VFA) was substantially higher in osteopenic patients, even though the difference was not significant (191 ± 126 vs. 139 ± 90; *p* = 0.070, Table1). In line with these findings, patients age and VFA were significantly associated with BMD using the Spearman ‘s correlation coefficient and corresponding correlations plots (r =  − 0.445, *p* = 0.000; r = 0.246, *p* = 0.014, Fig. 2). Detailed patient characteristics are displayed in Table 1.

### Perioperative outcome and osteopenia

In terms of perioperative outcomes, no difference was detected between the osteopenic and non-osteopenic subcohorts. Despite the lack of statistical significance, there was a tendency towards an increased perioperative morbidity in osteopenic patients. Major postoperative complications (CD ≥ 3b) occurred in 21% of the osteopenic patients and in 9% of non-osteopenics (*p* = 0.139, Table 2). The distribution of major morbidity is demonstrated in Table 3. Similar, CCI was higher but not significantly different in patients with osteopenia (24 ± 31 vs. 17 ± 25, *p* = 0.381, Table 2). In line with the findings above, mean hospital stay was 5 days longer in osteopenic patients but did not differ significantly (16 ± 15 vs. 11 ± 7, *p* = 0.103, Table 2), likewise the estimated procedural costs (14.2 ± 7.8 vs. 12.0 ± 6.8 TEuro, *p* = 0.147). Need of intraoperative FFP and RBC transfusion was similar between the groups (2.1 ± 2.5 vs. 2.1 ± 2.9 units *p* = 0.826; 1 ± 1.7 vs. 1.1 ± 1.9 units *p* = 0.906, Table 2). Five patients (15%) with normal BMD and 9 (13%) osteopenic patients died within the first 90-days following surgery (*p* = 0.909, Table 2, respectively).

**Table 2:** Perioperative outcome.

Characteristics	All patients	Osteopenia		*p* value
	n = 100	no n = 33	yes n = 67	
≥ CD3b complications^1^ including 90-day mortality n (%)	17	3 (9)	14 (21)	0.139
Hospital stay (days)	14 ± 13	11 ± 7	16 ± 15	0.103
Intraoperative RBC transfusion (units)	1 ± 1.8	1.1 ± 1.9	1 ± 1.7	0.906
Intraoperative FFP transfusion (units)	2 ± 2.7	2.1 ± 2.9	2.1 ± 2.5	0.826
CCI^3^	21 ± 89	17 ± 25	24 ± 31	0.381
Cost estimation (TEuro)^4^	13.5 ± 7.6	12.0 ± 6.8	14.2 ± 7.8	0.147

Values were given as mean ± standard deviation or absolute and relative frequencies (per cent). ^1^Refers to Clavien et al.^26^ ^3^Refers to Slankamenac et al.^27^ ^4^Refers to Staiger et al.^29^.

*CD* Clavien-Dindo classification, *ICU* intensive care unit, *RBC* red blood cell units, *FFP* fresh frozen plasma units, *CCI* Comprehensive Complication Index, *TEuro* thousand Euros.

**Table 3:** Perioperative complications.

Characteristics	Most important major complications during initial hospitalization
Bleeding	1
Cardiac	1
Pulmonary	3
Post-hepatectomy liver failure	2
Septic	10
Total	17

### Impact of osteopenia on long-term overall and disease-free survival

**Figure 3:**
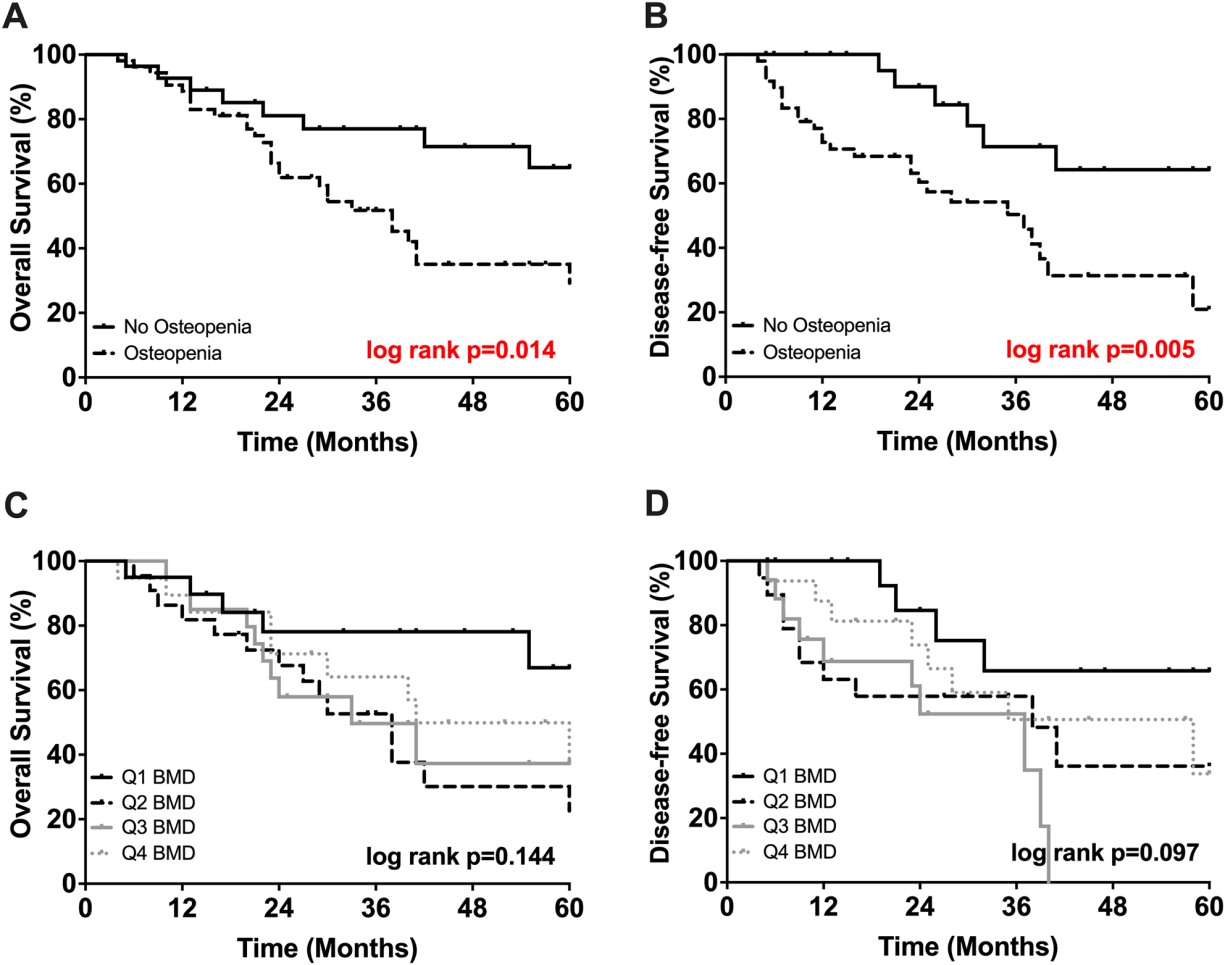
(**A**) 5-year survival of osteopenic and non-osteopenic patients. (**B**) Disease-free survival of osteopenic and non osteopenic patients. (**C**) 5-year survival divided by quartiles of bone density. (**D**) Disease-free divided by quartiles of bone density.

The median OS of all included patients in this study was 42 months with a median DSF of 37 months and a median follow-up period of 52 months. 5-year OS of osteopenic patients was significantly inferior when compared to those with normal BMD (29% vs. 65%, *p* = 0.014; Fig. 3, respectively). In line with the findings above, the probability of patient DFS at 5 years was significantly worse for patients with osteopenia compared with patients above the defined cut-offs of BMD (21% vs. 64%, *p* = 0.005; Fig. 3, respectively).

**Figure 4:**
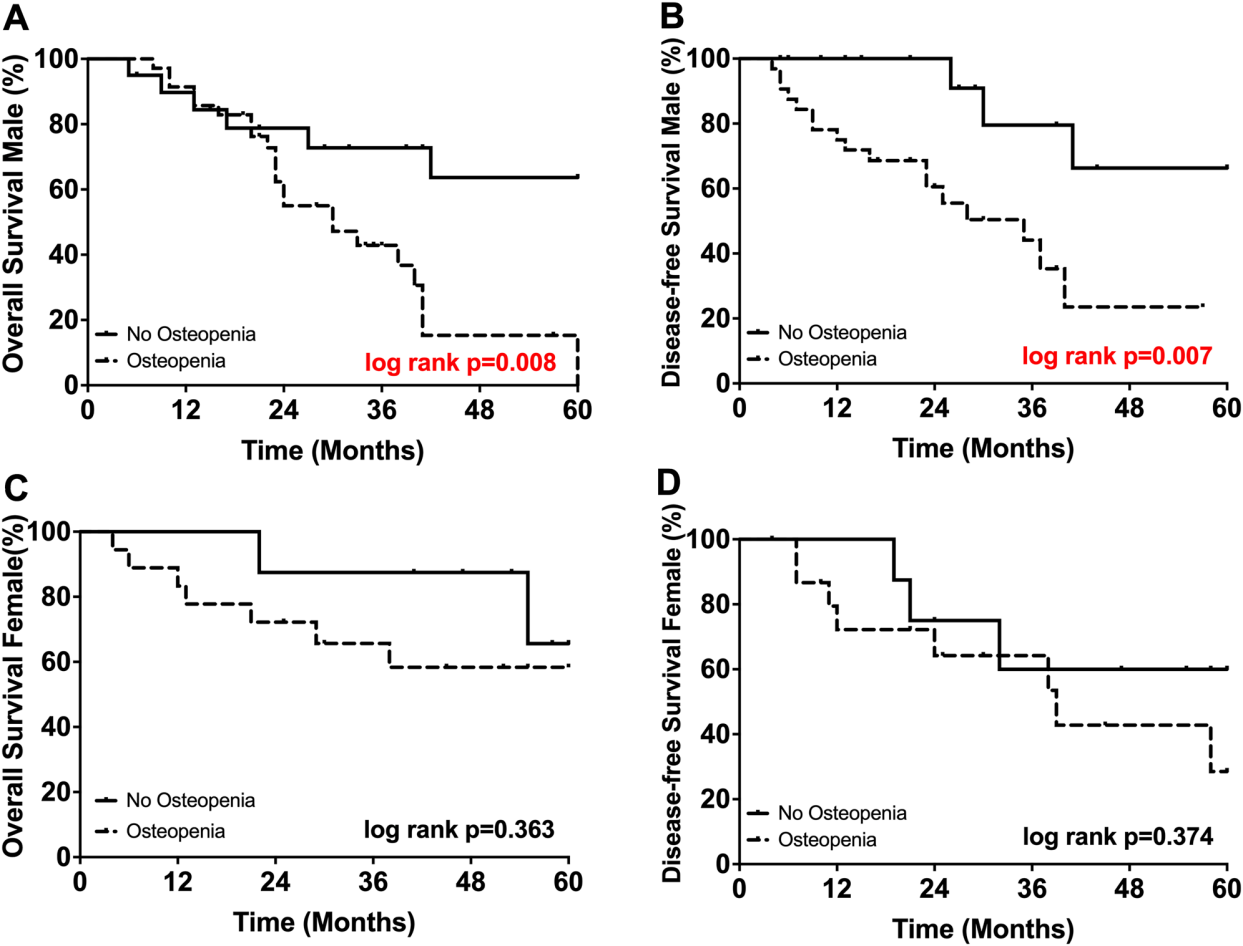
(**A**) 5-year survival of male osteopenic and non-osteopenic patients. (**B**) Disease-free survival of male osteopenic and non-osteopenic patients. (**C**) 5-year survival of female osteopenic and non-osteopenic patients. (**D**) 5-year survival of female osteopenic and non-osteopenic patients.

Further, due to the sex-related differences in BMD values we performed a subgroup analysis based on gender. In male patients suffering from osteopenia, 5-year OS was significantly impaired when compared with non-osteopenic males (0% vs. 64%, *p* = 0.008; Fig. 4). Disease-free survival in male patients was likewise significant impaired (24% vs. 66%, *p* = 0.007; Fig. 4). Interestingly, the findings above could not be confirmed in the female sub-cohort. While 5-year OS was largely comparable in female patients (58% vs. 66%, *p* = 0.374; Fig. 4), osteopenic females showed inferior DFS, even though the difference did not reach the levels of statistical significance (29% vs. 60%, *p* = 0.363; Fig. 4).

Finally, univariable Cox regression analyses revealed that pre-operative labMELD, intraoperative FFP and RBC transfusion and osteopenia were significantly associated with 5-year overall survival (Table 4). In the multivariable model, gender (HR 3.128 95% CI 1.159–8.444, *p* = 0.024, Table 4), pre-operative labMELD (HR 2.200 95% CI 1.030–4.699, *p* = 0.042, Table 4) and osteopenia (HR 7.743 95% CI 2.186–27.431., *p* = 0.002, Table 4) have been discovered to be independent predictors of inferior overall survival and demonstrated statistically significant results with meaningful hazard ratios (Table 4). Concerning DFS being outside the Milan criteria, AST, ALT, intraoperative FFP, negative R-0 status and osteopenia were found to be significantly associated with 5-year DFS in the univariable Cox regression analyses (Table 5). However, in the multivariable analysis, osteopenia lost its significant association with disease free survival while being outside the Milan criteria (HR 4.357 95% CI 1.493–12.714, *p* = 0.015, Table 5), intraoperative FFP transfusion (HR 3.693 95% CI 1.515–9.003, *p* = 0.004, Table 5) and not R0 resection (HR 3.356 95% CI 1.223–9.206, *p* = 0.019, Table 5) were still associated with disease free survival.

**Table 4:** Uni- and multivariable Cox regression analysis for overall survival.

	Univariable analysis		Multivariable analysis	
	Hazard-ratio (95% confidence interval)	*p value	Hazard-ratio (95% confidence interval)	*p* value
Age ≥ 65 years	0.992 (0.548–2.154)	0.802		
BMI ≥ 25	0.858 (0.494–1.490)	0.586		
Sex male	2.005 (0.939–4.281)	0.072	**3.128 (1.159–8.444)**	**0.024**
ASA ≥ 3	1.465 (0.800–2.684)	0.216		
Cirrhosis yes	1.468 (0.843–2.557)	0.175		
Preoperative labMELD ≥ 8	**2.121 (1.046–4.300)**	**0.037**	**2.200 (1.030–4.699)**	**0.042**
Outside milan criteria yes	2.020 (0.837–4.876)	0.118		
Preoperative AFP ≥ 10 (µg/l)	1.187 (0.396–3.558)	0.760		
Preoperative AST ≥ 40 (U/l)	1.425 (0.707–2.873)	0.323		
Preoperative ALT ≥ 40 (U/l)	1.448 (0.776–2.704)	0.242		
Preoperative Albumin ≤ 40 (g/l)	1.438 (0.667–3.102)	0.354		
Largest tumor diameter ≥ 50 mm	1.800 (0.882–3.769)	0.119		
Preoperative TACE yes	1.320 (0.317–5.498)	0.703		
Preoperative PVE yes	1.734 (0.617–4.877)	0.297		
Intraoperative FFP yes	**2.054 (1.056–3.961)**	**0.034**	1.499 (0.600–3.748)	0.386
Intraoperative RBC yes	**2.700 (1.33–5.469)**	**0.002**	1.740 (0.584–5.189)	0.320
Extended resection yes	1.309 (0.697–2.461)	0.402		
Vascular Reconstruction yes	0.960 (0.131–7.049)	0.968		
Duration Surgery ≥ 210 min	0.885 (0.508–1.542)	0.667		
Not R0-resection yes	1.977 (0.898–4.354)	0.091	2.103 (0.842–5.254)	0.111
Postoperative Complications (CD ≥ 3a^1^)	1.381 (0.680–2.806)	0.371		
Osteopenia (BMD) yes	**2.589 (1.173–5.715)**	**0.019**	**7.743 (2.186–27.431)**	**0.002**

Results of the Cox regression analysis were given as Hazard-ratios with 95% confidence interval.

^1^Refers to Clavien et al.^26^.

*BMI* Body mass index; *ASA* American Society of Anesthesiologists; *MELD* model for end-stage liver disease; *AFP* alphafetoprotein; *AST* Aspartate aminotransferase; *ALT* Alanine aminotransferase; *TACE* Transarterial chemoembolization; *PVE* Portal venous embolization; *FFP* Fresh frozen plasma; *RBC* Red blood cell unit; *CD* Clavien-Dindo classification; *BMD* Bone mineral density.

Significant values are in [bold].

**Table 5:** Uni- and multivariable Cox regression analysis for disease-free survival.

	Univariable analysis		Multivariable analysis	
	Hazard-ratio (95% Confidence Interval)	*p value	Hazard-ratio (95% Confidence Interval)	*p* value
Age ≥ 65 years	0.632 (0.335–1.193)	0.157		
BMI ≥ 25	1.004 (0.532–1.894)	0.991		
Sex Male	1.126 (0.585–2.168)	0.722		
ASA ≥ 3	1.005 (0.529–1.909)	0.988		
Cirrhosis yes	1.397 (0.741–2.631)	0.301		
Preoperative labMELD ≥ 8	1.395 (0.716–2.717)	0.328		
Outside Milan criteria yes	**4.283 (1.151–12.111)**	**0.006**	**4.357 (1.493–12.714)**	**0.015**
Preoperative AFP ≥ 10 (µg/l)	1.415 (0.587–3.412)	0.440		
Preoperative AST ≥ 40 (U/l)	**2.274 (1.147–4.509)**	**0.019**	1.618 (0.522–5.020)	0.405
Preoperative ALT ≥ 40 (U/l)	**2.154 (1.022–4.541)**	**0.044**	2.199 (0.700–6.905)	0.177
Preoperative Albumin ≤ 40 (g/l)	1.290 (0.628–2.648)	0.488		
Largest tumor diameter ≥ 50 mm	1.672 (0.853–3.279)	0.646		
Preoperative TACE yes	0.744 (0.101–5.464)	0.772		
Preoperative PVE yes	3.201 (0.960–10,677)	0.058	1.681 (0.188–15.048)	0.642
Intraoperative FFP yes	**2.025 (1.076–3.809)**	**0.029**	**3.693 (1.515–9.003)**	**0.004**
Intraoperative RBC yes	1.818 (0.886–3.728)	0.103		
Extended Resection yes	1.272 (0.621–2.604)	0.511		
Vascular Reconstruction yes	0.691 (0.091–4.893)	0.691		
Duration Surgery ≥ 210 min	0.610 (0.318–1.171)	0.137		
Not R0-Resection yes	**2.404 (1.168–4.947)**	**0.017**	**3.356 (1.223–9.206)**	**0.019**
Postoperative Complications (CD ≥ 3a^1^)	0.719 (0.331–1.563)	0.406		
Osteopenia (BMD) yes	**2.480 (1.169–5.260)**	**0.018**	2.053 0.793–5.314)	0.138

Results of the Cox regression analysis were given as Hazard-ratios with 95% confidence interval.

^1^Refers to Clavien et al.^26^.

*BMI* Body mass index; *ASA* American Society of Anesthesiologists; *MELD* model for end-stage liver disease; *AFP* alphafetoprotein; *AST* Aspartate aminotransferase; *ALT* Alanine aminotransferase; *TACE* Transarterial chemoembolization; *PVE* Portal venous embolization; *FFP* Fresh frozen plasma; *RBC* Red blood cell unit; *CD* Clavien-Dindo classification; *BMD* Bone mineral density.

Significant values are in [bold].

## Discussion

The present study shows the value of BMD and associated osteopenia as a clinical risk-factor in predicting oncological outcomes following partial hepatectomy for HCC in a Western-European cohort. While there was no significant difference in terms of perioperative morbidity between osteopenic patients and those with normal BMD, the prognostic value of BMD seems to be accentuated in the long run.

HCC is an important oncological entity with a worldwide increasing incidence^25^. Tumor recurrence and impaired long-term survival following liver resection are remaining key problems in the treatment of HCC patients^23,24,31^. Identification of novel risk-factors associated with inferior outcomes is of utmost clinical importance to optimize preoperative selection of surgical candidates and better balance the operative risk with the expected survival benefit.

Bone mineral density is known to be the most frequently used parameter to characterize the loss of bone mass and an important morphological component of patient frailty^32^. Although, DXA is the gold standard method in the diagnostics of osteopenia and osteoporosis, a growing body of evidence supports the use of radiation attenuation values of the trabecular bone based on routine staging CT-scans in oncological patients^22,33,34^.

While Sharma et al. were the first to explore the association between impaired BMD and HCC prognosis in a liver transplant setting, the Japanese group of Miyachi et al. has recently found an association between osteopenia and poor long-term outcome after partial hepatectomy for HCC^15,16^. Both groups used a general BMD cut-off of 160 HU to define osteopenia. However, due to a well-documented gender-specific difference in BMD values, the use of a non-gender specific cutoff was an important limitation of these previous two studies. Therefore, in our study we decided to implement sex-specific cut-offs for osteopenia which was similar to the strategy used recently by Sharshar et al. in a Japanese cohort of patients with pancreatic cancer^22,35^. While we used the well-established and frequently described cut-off of 160 HU for men, we chose a median-based cut-off of 175 HU for female patients^16^.

Using these cut-off values, we could show that osteopenic patients had significantly worse OS and DFS and osteopenia was identified as an independent risk-factor for inferior OS in our multivariable model. This is in line with the above-mentioned previous studies from Asian cohorts^15,16^. However, osteopenia was significantly associated with inferior DFS in the univariable Cox regression analysis, it could not be confirmed as independent risk-factor for inferior DFS in the multivariable model.

Next, we carried out a gender-specific subgroup analysis for overall and recurrence-free survival. While osteopenic male patients presented with a significantly inferior OS, this difference was not present in the female sub-cohort. Although, this gender-specific difference cannot be explained completely using our data, these findings are in line with those of Miyachi et al.^16^. In this Japanese study, these discrepancies were explained by the higher age of female patients with a more prominent age-related bone loss. However, in our present cohort, female patients were actually younger than males (median 63 vs. 69 years). Another possible explanation why BMD failed to stratify our female sub-cohort into high and low-risk groups may lie in the relatively low sample size of the female sub-cohort.

Various patient-related factors are known to influence BMD. These include for example race and menopausal status. As the study was carried out in a Western European hospital, the examined cohort was relatively homogenous concerning race. In line, most female patients were post-menopausal with only 3 females younger than 58 years.

Although, the mechanisms behind the association of bone loss and low BMD in the surgical and oncological setting remains to be fully elucidated^16,36,37^. A possible explanation for bone loss might be a paraneoplastic effect. Due to the impact of the tumor itself and its treatment on bone metabolism, cancer is known to be linked to bone loss. In this context, independently of sex or cancer type, the risk of osteoporosis is noticeably higher in patients suffering from cancer than in the general population^38^. It is assumed to be of immunological nature based on a relatively poorly understood cross-talk between bone, the immune system and the tumor itself^16,36^. This is supported by the observation that certain anti-resorptive drugs used in the treatment of osteoporosis also have significant anti-tumor effects via various immunological pathways. Inflammatory cytokines produced by tumors promote osteoclastogenesis^39–41^. Thus, cancer-related pro-inflammatory microenvironment accelerates bone loss. In this particular context, the association between cyclooxygenases, prostaglandin E2 as well as further mediators of cancer-related inflammation and accelerated bone density loss has been described before^42^.

Several studies have reported a correlation between reduced muscle mass (sarcopenia) and BMD. The group of Szulc et al. found that sarcopenia was associated with thinner bone cortices and a higher risk of falls in elderly male patients^43^. Although, in our study an association with SMI could not be confirmed, we found a highly significant negative correlation with patient age and VFA. No correlation was detected between the other analyzed BC parameters and BMD. Thus, the findings are partially in line with a recent publication by Sharshar et al. who reported a strong correlation between BMD and patient age, VFA and myosteatosis (IMAC and psoas muscle index) in an Asian cohort undergoing surgery for pancreatic cancer^22^.

Correlation of BC parameters and perioperative outcome has been described in various clinical conditions including chronic liver disease and OLT^3,9,44^. In our cohort, patients suffering from osteopenia developed more major complications (CD > 3b) although this difference was not significant. Nonetheless, CCI was higher and as a result, osteopenic patients stayed longer in hospital and the estimated procedural costs were slightly higher. Even though no statistically difference was found in terms of postoperative morbidity, presumably due to our relatively small sample-size, it can be assumed that osteopenic patients may present with an increased risk of developing complications when undergoing liver surgery for HCC than those with normal BMD. This should be assessed further in future studies.

Certain limitations of this study should be acknowledged here. First, it is important to reflect whether osteopenia cutoffs utilized in our analysis were adequate to properly identify patients at risk for poor outcomes. It is known that not only age, nutrition status, sex but also race and other specific factors related to this particular cohort and tumor-entity might strongly affect BMD values and their distribution^42^. These confounding factors could not be addressed properly in this retrospective dataset and should be explored further in prospective clinical trials with controlled data collection. Second, preoperative CT images used for BMD measurement were taken at various time points as part of the clinical routine and were analyzed in a retrospective and uncontrolled fashion. We could not explore longitudinal changes of BMD either, due to the limited availability of follow-up CT scans.

Notwithstanding these limitations, to the best of our knowledge, this study is the first report to evaluate the value and limitations of osteopenia as a risk factor of clinical outcomes following curative-intent liver surgery for HCC in a western-European single-center cohort. The use of BMD as a prognostic marker lies in its simplicity. Although, it may never replace the subjective assessment of “fitness for surgery” by an experienced hepatobiliary surgeon or hepathologist, in combination with other body composition and frailty parameters it may serve as a useful clinical tool to improve pre-operative patient selection in HCC. Further prospective clinical trials are warranted to validate these findings and assess functional components of frailty and BC at the same time. Most pre-habilitation and enhanced-recovery programs are currently focusing on the muscle compartment, physical function measured predominantly by parameters of muscle function and fitness^5^. In the context of the present findings, an interesting direction of future research would be to develop therapeutic and pre-habilitation approaches directed specifically towards frail osteopenic patients.

## Data Availability

All relevant data were reported within the manuscript. Further supporting data will be provided upon written request addressed to the corresponding author.

## Abbreviations

ALT: Alanine aminotransferase
ASA: American society of anesthesiologists
AST: Aspartate aminotransferase
BC: Body composition
BMD: Bone mineral density
BMI: Body mass index
CCI: Comprehensive complication index
CD: Clavien-Dindo classification
CI: Confidence interval
CT: Computed tomography
CUSA: Cavitron ultrasonic surgical aspirator
FFP units: Fresh frozen plasma units
GCP: Good clinical practice
GGT: Gamma glutamyltransferase
HCC: Hepatocellular carcinoma
HU: Hounsfield unit
ICU: Intensive care unit
L3: Third lumbar level
MELD: Model of end-stage liver disease
OR: Odds-ratio
PVE: Portal vein embolization
POD: Postoperative day
TACE: Transarterial chemoembolization
TARE: Transarterial radioembolization
RA: Radiation attenuation
RBC units: Red blood cell units
SE: Standard error
SFA: Subcutanous fat area
SMI: Skeletal muscle index
TEur: Thousand Euros
UH-RWTH: University hospital of the RWTH university
UICC: Union for international cancer control
VFA: Viseral fat area
VSR: Visceral-to-subcutaneous fat ratio
